# Effects of a Multicomponent Mycotoxin Detoxifying Agent on Health and Performance of Weaned Pigs Under Combined Dietary Exposure to Deoxynivalenol (DON) and Zearalenone (ZEN)

**DOI:** 10.3390/toxins17030146

**Published:** 2025-03-19

**Authors:** Jog Raj, Panagiotis Tassis, Klaus Männer, Hunor Farkaš, Zdenka Jakovčević, Marko Vasiljević

**Affiliations:** 1 Patent Co, DOO., Vlade Ćetkovića 1A, 24211 Mišićevo, Serbia; hunor.farkas@patent-co.com (H.F.); zdenka.jakovcevic@patent-co.com (Z.J.); marko.vasiljevic@patent-co.com (M.V.); 2 Farm Animals Clinic, School of Veterinary Medicine, Aristotle University of Thessaloniki, 54627 Thessaloniki, Greece; 3 Institute of Animal Nutrition, Freie Universität Berlin, Königin-Luise-Str. 49, 14195 Berlin, Germany; klaus.maenner@fu-berlin.de

**Keywords:** weaned pigs, deoxynivalenol, zearalenone, mycotoxins, health, inflammatory biomarkers, mycotoxin residues, detoxifying agent

## Abstract

The aim of the present study was to evaluate the efficacy of a multicomponent mycotoxin detoxifying agent (MMDA, MYCORAID, Patent Co, Mišićevo, Serbia) in weaned pigs receiving contaminated feed with deoxynivalenol (DON) and zearalenone (ZEN). In total, 168 pigs were equally allocated in four experimental groups from day 25 to day 66 of age. The T1 group received feed without mycotoxins or MMDA. The pigs in group T2 received contaminated feed (CF) with 1.5 mg of DON/kg feed and 0.9 mg of ZEN/kg feed for the first two weeks and 1.2 mg of DON/kg feed and 0.9 mg of ZEN/kg feed for the rest of the trial period, without the addition of MMDA. Groups T3 and T4 received the CF with the addition of 1.5 g of MMDA/kg feed (T3), or 3 g of MMDA/kg feed (T4). Performance parameters, stress, and inflammatory biomarkers, as well as mycotoxin residues in liver, kidney, and muscle tissue were assessed. The results demonstrated improved average daily gain (ADG) and feed conversion ratio (FCR) along with reduced DON residues in kidney samples in groups T3 and T4 when compared with the T2 group. Although a typical dose–response relationship was not present in all parameter alterations, the results of the study proved the efficacy of the test product with improved growth performance and reduced mycotoxins absorption under the concurrent DON and ZEN exposure conditions and supported its use as a mitigating tool against mycotoxicosis under field conditions.

## 1. Introduction

Mycotoxins are toxic fungal secondary metabolites frequently detected as contaminants of grains. They induce toxic effects in farm animals, causing distress and reduced productivity [1]. Mycotoxins produced by *Fusarium* species are among the most frequently detected in grains worldwide [2,3]. Among various *Fusarium* mycotoxins, pigs are significantly susceptible to the effects of deoxynivalenol (DON) and zearalenone (ZEN) [4,5]. The European Commission has set maximum recommended contamination levels of 0.9 mg of DON/kg feed, 0.1 mg of ZEN/kg feed for piglets and gilts, and 0.25 mg of ZEN/kg feed for sows and fattening pigs, respectively [6].

DON is a member of the trichothecene family of mycotoxins produced typically by *Fusarium graminearum* and *Fusarium culmorum* [7,8]. Significant absorption of the toxin takes place in the upper digestive system, whereas its systemic bioavailability ranges between 52.7% and 100% after oral exposure [9]. DON is mainly excreted through urine, thus a lower rate of metabolization is indicated [10]. After ingestion, DON is metabolized by intestinal microbes to de-epoxy-DON (DOM-1), which can be found in plasma or excreta [11]. DON binds to the ribosomal 60S unit, inducing ribotoxic stress, which leads to reduced protein synthesis, whereas alterations in the mitochondrial structure and functioning have also been reported. Among various toxic effects (neurotoxicity, reprotoxicity, and immunosuppression), DON significantly affects the gastrointestinal tract (GIT). DON induces a reduction in goblet cell production, which affects the expression of tight junction proteins, such as claudins [12], and thus negatively affects the intestinal barrier and the intestinal microbiome inducing intestinal dysbiosis [13]. Previous in vivo studies in swine have also reported its emetic potency (12 and 20 mg/kg feed in growing pigs), reduction in appetite, and decreased weight gain (1–2 mg/kg feed in growing pigs) [14].

ZEN is a phenolic resorcyclic acid lactone mycotoxin which acts by binding estrogen receptors (ERs), with a stronger affinity to ER-α compared to ER-β. ZEN absorption reaches 61–85%, but only 1.8% of the parent toxin is systemically available due to the extensive first-pass metabolism in the intestine and liver [15]. Its metabolic pathway after oral exposure includes rapid absorption from the intestine and extensive liver phase I and II biotransformations. Major reductive metabolites of ZEN are α-zearalenol (α-ZEL—main form in swine) and β-zearalenol (β-ZEL), whereas α- and β-zearalanol (α-ZAL and β-ZAL, respectively) and zearalanone (ZAN) are considered as metabolites of minor importance in swine [5]. ZEN and metabolites are mainly excreted through urine (between 14 and 77% of ZEN dose can be recovered in porcine urine) [9]. ZEN toxicosis in gilts and sows is characterized by reddening, hyperemia, and edematous swelling of the vulva, enlargement of the uterus and mammary glands with ovarian cysts presence. Moreover, vaginal or rectal prolapse, as well as atrophy of the testes and detrimental effects on the kinetics and viability of boar spermatozoa, have been reported [16]. In addition, ZEN can induce oxidative stress, decrease nutrient digestibility, or reduce growth rate [17,18].

The presence of mycotoxin mixtures in swine feed is a common finding globally, with DON and ZEN’s concurrent presence as one of the most frequently observed combinations [2]. Previous research efforts have provided evidence of their importance in terms of health and performance effects in pigs, as well as their interactions when ingested together by swine [2,19]. After four weeks of exposure to 8 mg/kg DON and 0.8 mg/kg ZEN, decreased body weight (BW), average daily feed intake (ADFI), feed conversion rate (FCR), and immunoglobulin IgG and IgM in serum have been reported in six-week-old pigs [20]. Jia et al. [21] also observed that piglets co-exposed to both toxins showed significantly decreased body weight gains and ADFI, indicating a synergistic negative impact on intestinal function and subsequent systemic inflammation. On the other hand, Thapa et al. [22] suggested a range from synergistic to antagonistic toxic effects after co-exposure to DON and ZEN, whereas Le Sciellour et al. [23] reported on the transient effects of combined toxins exposure on the fecal microbiota of swine. Synergism after combined DON and ZEN exposure has been reported by Tassis et al. [24], in particular boar semen motility and viability parameters in vitro, whereas evidence of immune response alterations in either an additive or antagonistic way has also been demonstrated under concurrent dietary exposure to great concentrations of DON and ZEN [14]. Therefore, it seems that both toxins, when present together in swine feed, are capable of inducing a variety of dose and time-dependent negative health and performance effects in swine based on their mode of action and interaction effects.

A common approach for the mitigation of mycotoxin effects in animal production includes feed additives such as binders. However, it should be noted that DON has a low affinity to binders due to its structurally low polarity. Novel products containing microorganisms (e.g., *Bacillus licheniformis* YB9, *Bacillus subtilis* ASAG 216, and *Lactobacillus rhamnosus* SHA113) with the ability to degrade DON into less toxic compounds such as DOM-1, 3-keto-DON [25], 3-epi-DON [26], and 3-epi-DOM-1 [27] are gaining increased interest in recent studies, along with fungi and yeast (*Aspergillus tubingensis*, *Aspergillus oryzae*, *Rhizopus oryzae*, and *Saccharomyces pastorianus*) acting as DON degraders [10,28,29,30]. Mycotoxin-detoxifying agents containing multiple components have shown more benefits in comparison to those with single components [31], and such agents, including combinations of adsorbents, health stimulants, and detoxifiers, seem to induce beneficial results in cases of dietary exposure to multiple mycotoxins in swine [3].

The aim of the present study was to evaluate the efficacy of a multicomponent mycotoxin detoxifying agent (MMDA, MYCORAID, Patent Co, Mišićevo, Serbia) supplemented at 1.5 and 3 g/kg feed into concurrent deoxynivalenol (DON)- and zearalenone (ZEN)-contaminated diets of weaned pigs from 25 to 66 days of age.

## 2. Results

### 2.1. Clinical Signs, Morbidity, and Mortality

The overall mortality and medication rate of piglets during the 42 d experimental period amounted to 1.19% (two animals in total), and 3.57% (six animals in total: two animals received injectable amoxicillin due to respiratory disorders and four animals received injectable meloxicam due to claw injury), respectively. Due to the low mortality and medication rate, a treatment-related correlation could not be established. The remaining piglets were without visual clinical signs throughout the 42 d experimental period. Therefore, the dietary exposure of DON and ZEN did not produce acute clinical symptoms. Regarding fecal scoring, the mean values of all groups indicated predominantly well-formed feces soft or firm to cut; thus, detrimental effects of mycotoxin contamination or protective effects of MMDA on fecal consistency could not be demonstrated.

### 2.2. Performance Parameters

Performance parameter alterations of post-weaning piglets recorded from 25 to 66 days of age (day 0 to day 42 on trial) are presented in Table 1. Feeding DON- and ZEN-contaminated diets without MMDA (T2 group) resulted in a significantly reduced overall BWG (start to end of the study), whereas the addition of MMDA at 1.5 g/kg feed (T3 group) and 3 g/kg feed (T4 group) induced a significant improvement of BWG. That improvement in the T4 group “restored” the mean value of BWG to one similar to the control group (T1) level for the total trial period. In addition, the overall mean feed intake of the T1 group piglets amounted to 38.71 kg or 922 g per day, respectively, whereas significantly lower results were found for the piglets in the T2 group (reduction by 7.9%). Feed intake was improved in T4 but not in the T3 group for the overall trial period when compared with T2. Significantly, FCR and ADG values of both the T3 and T4 groups were improved in comparison with the T2 group for the total trial period, demonstrating an improved overall growth performance. Effect size (eta squared, η^2^) for the overall trial period analysis was for BWG and ADG η^2^ = 0.88, whereas for FCR η^2^ = 0.711.

### 2.3. Hematological and Plasma Biochemical Parameters

The results of health-relevant blood indices determined in selected piglets at the study end (d 66 of age) are summarized in Table 2. The results of the blood analysis at the end of the 42 d feeding phase were within the physiological range for healthy post-weaning piglets. However, significantly reduced haptoglobin and MDA values, along with reduced AST were observed in T4 group animals, when compared with the T2 group. The T3 group mean values of MDA were also improved in comparison with T2 group animals. Furthermore, haptoglobin levels were reduced in the T4 group when compared to the T2 group.

### 2.4. Stress- and Inflammatory-Biomarkers in Feces

The differences among mean values of calprotectin (effect size η^2^ = 0.289) and cortisol (effect size η^2^ = 0.586) as fecal stress and inflammatory biomarkers between trial groups at the end of the study period are summarized in Table 3. The T2 group animals showed increased cortisol and calprotectin concentrations in feces in comparison with the control group. The application of MMDA only at 3 g/kg feed (T4 group) reduced cortisol level, when compared with the T2 group.

### 2.5. Mycotoxin Residues in Tissues

The results of the mycotoxin residue analysis are presented in Table 4. The recovery of DON in the T2 group samples showed the highest values in the kidneys, whereas the DON and ZEN levels in the selected muscle tissue samples of all groups were below the detection limit. DON and ZEN metabolites residues in all tissue samples were below the detection limit. However, a significant reduction in DON residue levels in kidneys was detected in both T3 and T4 group samples in comparison with the T2 group (−44.7% in the T4 group). A numerical reduction in DON residues in liver samples of MMDA-treated groups when compared with the T2 group was also observed. ZEN contents in liver and kidneys of T3 and T4 groups were slightly reduced when compared to the T2 group. The effect size for DON residues in kidneys was η^2^ = 0.662, whereas for ZEN residues, it was η^2^ = 0.753. In liver samples, the effect size for DON residues analysis was η^2^ = 0.728, and for ZEN residues, it was η^2^ = 0.770. Supplementary Figures S1–S4 demonstrate chromatograms of DON and ZEN residues analysis in kidneys and liver samples per trial group.

## 3. Discussion

The results of the present study provide evidence of the ability of the MMDA to induce improvements in primary performance parameters and reduce DON residues in kidneys, as well as reduce oxidative and inflammatory biomarkers and induce alterations in plasma biochemical parameters, which imply a liver protective effect in pigs in cases of long-term ingestion of contaminated feed with DON and ZEN. The positive effects of MMDA introduction in feed were more pronounced and significant in the group that received 3 g of MMDA/kg feed.

Previous in vivo studies from our group [32,33] on the efficacy of MMDA under combined mycotoxin exposure conditions in weaned pigs have also proven its detoxifying properties against combined exposure to ZEN (0.35 mg of ZEN/kg feed) and T2 (0.5 mg of T-2/kg feed), or ZEN (approximately 1 mg of ZEN/kg feed) and ochratoxin A (0.5 mg of OTA/kg feed). In both studies, the residues of ZEN [32] or ZEN and metabolites a- and β-ZEL [32] were significantly reduced in liver and kidney samples, either in both groups receiving MMDA (i.e., 1.5 and 3 g of MMDA/kg feed) [32], or only in the group that received the highest MMDA dosage level (i.e., 3 g of MMDA/kg feed) [33]. Such a reduction in ZEN residues in the present study was present only as a numerical reduction of approximately 9.1% in liver samples and 15.4% in kidney samples of the T4 group when compared with the T2 group, whereas the respective reduction in the T3 group was smaller. The differences in the present results with the above-mentioned studies most probably rely on a possible interaction between DON and ZEN, which could lead to the altered affinity of MMDA to ZEN in the present study. On the other hand, ZEN metabolite residues in tissues were below detection limits in the present study. Quite similarly, in a study by Gajęcka et al. [34] with low DON (approximately 300 μg/kg feed) and greater ZEN concentration (approximately 1000 μg/kg feed) in the feed of weaned gilts for 42 days, ZEN metabolites in intestinal and liver tissues were also absent, whereas the carry-over factor of ZEN was absent or very low. The latter was attributed to a possible “physiological deficiency” of endogenous estrogens. The authors suggested that this assumption is validated by the absence of both ZEN metabolites in intestinal tissues and the significant increase in ZEN concentration in the duodenum, jejunum, and liver [34].

The DON metabolic pathway is characterized by its absorbance in the proximal aspect of the small intestine and its significant bioavailability in pigs. The intestinal mucosa, the liver, and kidneys are major sites of DON phase II metabolism, which leads to DON and DOM-1 conjugates. As already reported, the greatest carry-over factor for DON is expected in the kidneys, followed by the liver [11]. A maximum carry-over of 0.008 into the liver or 0.034 into the kidneys has been reported for the sum of DON and DOM-1 in previous studies on pigs fed with DON-contaminated diets [11]. That explains the significant difference in the DON residue levels detected among the liver and kidney samples in our study. On the other hand, the reduction in mean DON concentration in kidneys reached 43% in the T3 group and 46.3% in the T4 group when compared with the mean values of the T2 group, providing evidence of the reduced bioavailability of DON in MMDA-treated groups, which can be attributed to the adsorbing and detoxifying effects of MMDA. The significant effect size for DON (greater than η^2^ = 0.14, as suggested by Cohen [35]) suggests the aforementioned detoxifying effect of the test product in reducing DON residues in the tissues of animals receiving the test product. The DON de-epoxidation process toward DOM-1 has been reported to increase from the proximal to the distal part of the digestive tract [11], whereas the carry-over of DOM-1 comes from its gut-derived absorption. The absence of DOM-1 residues in tissues in the present study has also been demonstrated in previous studies [36,37,38], supporting a very limited carry-over of DOM-1 to pig tissues after ingestion of contaminated feed. The absence of DOM-1 in tissues in our study can be explained by the combination of reduced DON uptake due to the adsorbing and detoxifying properties of MMDA, along with almost complete DON absorption in the upper digestive tract before microbial de-epoxidation occurs [39].

Under oxidative stress conditions, the level of MDA, as the final product of lipid peroxidation, is expected to increase [40]. Increased MDA levels have been reported after DON and ZEN exposure in vitro in porcine splenic lymphocytes [41], as well as after dietary exposure in vivo [42]. Moreover, acute phase proteins (APPs) are liver-derived plasma proteins, which have concentrations that can alter as a response to internal or external challenges affecting homeostasis, such as infection, tissue injury, and trauma [43,44]. Positive APPs such as haptoglobin, increase after such challenges. The results of the present study demonstrated a reduction in oxidative stress and inflammatory biomarkers in MMDA-treated pigs after concurrent DON and ZEN ingestion via feed. Even though within normal limits, the reduction in MDA levels in the T3 and T4 groups, as well as the reduction in haptoglobin in the T4 group, clearly demonstrate the outcome of detoxifying mechanisms due to the inclusion of MMDA in the diets of T3 and T4 group pigs against oxidative stress and inflammatory processes induced by DON and ZEN. Our findings agree with previous studies which provide evidence of the ability of *B. subtilis* ANSB01G to alleviate ZEN-induced oxidative stress in gilts [40] or montmorillonite clay to reduce similar stress indices in weaned gilts [17], or S. cerevisiae to reduce inflammation and oxidative stress and increase cell survivability in DON-challenged pigs through the reversing of particular inflammation signaling pathways, such as NF-κB and p38 MAPK [45]. Moreover, silymarin has also been characterized by hepatoprotective, antioxidant, and anti-inflammatory properties. Antioxidant properties of silymarin include various mechanisms, such as direct free radical scavenging, the prevention of free radical formation (through the inhibition of specific enzymes), supporting the integrity of the electron-transport chain of mitochondria in stress conditions, sustaining the cell’s optimal redox status (through the activation of antioxidant enzymes and non-enzymatic antioxidants), and activating an array of vitagenes [46]. A combination of the above-mentioned mechanisms of the MMDA ingredients in the present study has probably been responsible for the beneficial effects observed on mycotoxin-induced oxidative stress.

Moreover, the numerical reduction in calprotein levels in both MMDA-fed groups, but especially in the T4 group, when compared with the T2 group further supports the observation of a possible counteracting reduction in inflammatory processes introduced by DON and ZEN due to the ingestion of MMDA. Fecal calprotein has been widely used to assess the extent of intestinal inflammation in human medicine [47], whereas elevated levels in pigs have been associated with colitis [48]. Considering that DON can cause intestinal damage and intestinal flora imbalance [49], even at the low contamination level of 0.9 mg of DON/kg feed [50], our results in MMDA-treated animals with reduced fecal calprotein levels, further support the detoxifying effect of the test product in terms of reduced intestinal inflammation in pigs after long term dietary exposure to DON and ZEN. Additionally, cortisol levels in blood or saliva have been recognized as a stress indicator in swine [51,52], and increased levels have been detected due to different stressors, such as shipping, altered ambient temperature, and social stress [53]. Such a neuroendocrine response to stress involves the hypothalamic–pituitary–adrenal axis, which is implicated in metabolism, reproduction, and the development of immunocompetence in pigs [49]. A previous study suggested a connection between stress and reduced productivity, as observed with reduced feed intake and growth rate [54]. Therefore, the significant reduction in cortisol levels observed in our study could be well associated with the observed improved performance parameters such as ADFI (T4 vs. T2 group for the overall trial period), BWG, and ADG (both T3 and T4 vs. the T2 group for the overall trial period) in animals receiving MMDA.

One of the DON predominant effects is the reduction in protein synthesis [3], whereas both DON and ZEN are capable of inducing liver dysfunction [55,56,57] accompanied by serum biochemistry alterations such as the increase in liver enzyme activities [17,21]. On the other hand, increasing evidence supports the important role of oxidative stress as a key mechanism of hepatotoxicity, whereas mycotoxins are able to generate reactive oxygen species, which induce lipid and protein oxidation [46]. Our findings of improved total protein levels and reduced AST values in the T4 group support a possible liver protective effect of the test product’s ingredients at the greatest dosage level. Such effects could be well connected with silymarin’s ability to protect against mycotoxin-induced hepatic TNF-alpha release and to restore the levels of the cytotoxic markers [58,59,60]. Moreover, previous studies have demonstrated also the ability of yeast additives to reduce liver damage after dietary exposure to DON and/or ZEN in pigs [61,62], whereas the protective effect of *Bacillus* probiotic species against liver damage attributed to DON and ZEN has already been demonstrated [21,55,63,64].

DON is able to reduce feed intake through various mechanisms, such as the modulation of local serotonin and the decrease of bowel movements [65,66]. Moreover, greater satiety signaling [67] after DON exposure has been demonstrated, along with the release of proinflammatory cytokines, and at high dosage levels, the induction of vomiting [68]. It has been suggested that the intestinal microbiota and the brain–gut axis play a crucial role in DON-induced growth inhibition, whereas a number of mechanisms involved in DON toxicity have been reported, such as the activation of oxidative stress, and the MAPK signaling pathway [69]. Moreover, growth retardation is attributed to DON-induced disruption of the intestinal barrier, reduced nutrient intake, and nutrient absorption [70]. On the other hand, although ZEN can induce inflammation and deleterious effects on intestinal morphology in pigs at low doses, its acute effect on pig growth and performance has been discussed as controversial [3]. Reduction in ADG and feed efficiency has been observed in a previous study with ingestion of 0.2–0.8 mg of ZEN/kg feed for 28 days in weaning gilts [71]. In another study with the inclusion of 1 mg of ZEN/kg feed ingestion for 24 days in weaned gilts, BW, ADG, and ADFI were increased [72]. Nevertheless, the synergic toxic potency of DON and ZEN has been previously reported in various tissues, such as their synergic inhibitory effect on cell proliferation of intestinal porcine cells [73]. On the other hand, DON and ZEN concurrent ingestion can be considered as a risk factor for subclinical inflammation in the small intestine [74], or as a potential disrupting factor of the defense mechanisms of the large intestine [75]. A recent study with piglets (3-week diet with approximately 1 mg of DON/kg feed and/or 265–269 mg of ZEN/kg feed) showed that co-exposed piglets had significantly lower BWG and ADFI, indicating that both toxins were acting synergistically to disrupt intestinal functions and cause systematic inflammation [21]. Our results demonstrated reduced BW and BWG in the T2 group. On the other hand, the observed improvement of BWG, ADG, and FCR in the T3 and T4 groups and BW only in the T4 group when compared with the T2 group in the overall study period should be highlighted as a clear indication of the ameliorating properties of MMDA (at both dosage levels) against the negative impact of DON and ZEN on growth and performance. Accordingly, the significant effect size observed for FCR, ADG, and BWG parameters for the total trial period suggests a significant impact of the test product on the improvement of these three productive parameters, whereas results support its practical implementation under field conditions with promising results.

The mixed contamination of swine feed with DON and ZEN is very common in grains used as swine feed globally and requires control measures which will eliminate the adverse effects of both toxins in pigs. Moreover, it becomes clear that detoxifying agents with multiple ingredients, which are able to alleviate mycotoxin mixtures, can play a significant role in protection against the mycotoxin menace in pig production. The limitations of the present study include the lack of testing of additional biological matrices, such as urine or feces for DON and ZEN biomarkers of exposure, the absence of a clear dose–response relationship among MMDA levels, and the alterations of all tested parameters. However, our findings support the detoxifying properties of MMDA under combined DON and ZEN dietary exposure in pigs. Proposed mechanisms of action of the test product against DON and ZEN feed exposure in pigs include a combination of the adsorbing capabilities of modified zeolite (clinoptilolite), along with the enzymatic activity and the cell wall adsorbing capabilities of *Bacillus subtilis*, *Bacillus licheniformis*, and *Saccharomyces cerevisiae* [76,77,78], which can reduce mycotoxins’ bioavailability and work along with the liver-protective, antioxidant, and anti-inflammatory effect of silymarin as a robust multicomponent mechanism. Such a symbiotic system of mycotoxin mitigating compounds has also been demonstrated in a study by Markowiak et al. [79], where the advantage of combining several bacterial strains (lactic acid bacteria) with *Saccharomyces cerevisiae* improved the degradation of OTA. On the other hand, it has been previously demonstrated that mycotoxin detoxifiers targeting DON often show a higher detoxifying capacity if multiple components are present both in vitro and in vivo [80].

In conclusion, the results of the present study provided evidence of the mitigating properties of a multicomponent product (MMDA) in pig feed against concurrent DON and ZEN exposure. The findings support the use of MMDA as part of a detoxifying program against dietary mycotoxin exposure under field conditions. Nevertheless, further in vivo evaluations of such feed additives with detoxifying properties with the use of novel biomarkers against dietary exposure to mycotoxin mixtures in swine should be promoted in future research efforts.

## 4. Materials and Methods

The trial was performed in accordance with the Animal Welfare Act of Germany, approved by the local state Office of Occupational Health and Technical Safety (Landesamt für Gesundheit und Soziales, LaGeSo, no. A 0439/17). Animals used in the study were raised and treated according to European Union Directive 2010/63/EU [81], covering the protection of animals used for experimental or other purposes and according to the recommendation of Commission 2007/526/CE [82] covering the accommodation and care of animals used for experimental and other scientific purposes. The trial was approved by the Ethical Committee of the Freie Universität Berlin with approval Nr A 439/17.

### 4.1. Animals and Diets

In total, 168 post-weaning barrows and gilts (Danbred x Duroc) at the age of 25 days were introduced in the study. The study was performed in an experimental farm in Denmark (DK 6360 Tinglev) and lasted until day 66 of age. The animals were randomly allocated into four treatment groups of 42 animals each, which were equally divided according to body weight, litter, and gender in seven pens/treatment with six piglets/pen. A double blind-method was used, in which the personnel handling the animals were not aware of the specific feed treatments/group, and the laboratory personnel were not aware of the allocation of animals and respective samples in each group. All trial animals had ad libitum access to feed and water throughout the study.

During the 42 d experimental period, two basal diets presented as mash feed (particle size: 1.5 to 3 mm) from 25 to 38 days of age (basal starter diet), and from 39 to 66 days of age (basal grower diet) were offered to the animals. The starter and grower diets were calculated to be iso-nutritive, meeting or slightly exceeding the nutritional requirements for weaned piglets, as recommended by the Society of Nutrition Physiology [83]. All diets were manufactured in a commercial feed mill and were formulated without added antibiotics, organic acids, polysaccharides, enzymes, yeast/egg products, porcine plasma, or zootechnical feed additives to avoid the potential confounding effect of these additives.

The basal ingredients for the diets were provided by the feed mill, MMDA, as well as maize spiked with DON and ZEN were supplied by the study sponsor (Patent Co, Mišićevo, Serbia). DON and ZEN mycotoxins were produced “in house” using a *Fusarium* sp. fungi. The Fusarium fungi were isolated from contaminated crops. The production of mycotoxins was performed in sterilized maize kernels at 28 °C for 35 days, with an initial moisture of 30%. After 5 weeks of incubation, the contaminated corn was placed in the drying oven at 105 °C for 3 days until it was completely dry. The material was then milled into a fine powder, and the mycotoxin content was analyzed using LC-MS/MS. The ingredients, premixes, and the calculated analyses of the experimental diets are presented in Table 5 and Table 6. The test product administered in the study was an MMDA containing modified zeolite (clinoptilolite), *Bacillus subtilis, Bacillus licheniformis, Saccharomyces cerevisiae* cell wall, and silymarin [32,33].

The animals in the T1 group received the control feed without the addition of mycotoxins or the test product. The pigs in group T2 served as positive controls and received contaminated starter feed with 1.5 mg of DON/kg feed and 0.9 mg of ZEN/kg feed for the first two weeks of the trial and contaminated grower feed with 1.2 mg of DON/kg feed and 0.9 mg of ZEN/kg feed for the rest of the trial period without the addition of MMDA. Group T3 received the above-mentioned contaminated feed as for the T2 group with the addition of 1.5 g of MMDA/kg feed, whereas the animals of group T4 also received the same contaminated feed with the addition of the 3 g of MMDA/kg feed.

The MMDA was added at the expense of Tixosil (>97% silicon dioxide; total amount of 0.3% in the control diet) in the diets of the T3 and T4 groups. DON and ZEN were added in the form of a maize-based premix (0.34%) in exchange for corresponding proportions of control maize. To conduct proper mixing, premixtures containing aliquots of basal diets (25 kg each), and the overall amounts of Tixosil and control maize (T1 group), Tixosil and DON/ZEN premix (T2 group), Tixosil and the MMDA as well as the DON/ZEN premix (T3 group), and the MMDA and DON/ZEN premix (T4 group) were produced by very gentle mixing and included the corresponding batches of starter and grower diets, respectively. The feed samples of each experimental diet were collected for analysis according to the EC Regulation 152/2009 [85].

### 4.2. Feed Analysis and Mycotoxin Residue Assessment of Tissue Samples

All experimental diets were ground to pass through a 0.25 mm screen before analysis. The laboratory measurements included Weender constituents and, additionally, starch, total sugars, calcium, phosphorus, and sodium. The analyses were conducted in accordance with the methods issued by the Association of German Agricultural Analytic and Research Institutes [86] (dry matter: VDLUFA III 3.1; crude protein: VDLUFA III 4.1.1 modified according to macro-N determination (vario Max CN); crude fiber: VDLUFA III 6.1.4; crude ash: VDLUFA III 8.1; crude fat: VDLUFA III 5.1.1; starch: VDLUFA III 7.2.1; total sugars: VDLUFA III 7.1.1; calcium: VDLUFA VII 2.2.2.6; phosphorus: VDLUFA VII 2.2.2.6; sodium: VDLUFA VII 2.2.2.6). Aflatoxins, DON, ochratoxin A, ZEN, and T2 levels in feeds were determined with LC-MS/MS in accordance with FB 558-03-IAC-R7 (LOD: 0.1 µg/kg), FB 534-02-R11 (LOD: 50 µg/kg), FB-535-04-R10 (LOD: 1 µg/kg), FB-547-01-R12 (LOD: 10 µg/kg), and FB-554-78-R7 (LOD: 25 µg/kg) [32,80].

The piglets used for blood and fecal sampling were euthanized by T61 (Intervet International GmbH, Unterschleißheim, Germany) after anesthesia using a combination of ketamine–hydrochloride (Ursotamin, Serumwerk Bernburg AG, Bernburg Germany) and azaperone (Stresnil, Janssen Pharmaceutica N.V., Beerse, Belgium). The liver, kidneys, and M. semitendinosus were excised and prepared (seven samples per organ and treatment). Afterward, the samples were packed into polyethylene bags and kept at −20 °C before being freeze-dried and subjected to mycotoxin analyses.

The DON and ZEN contents in liver, kidney, and muscle tissues were measured at the study sponsor’s laboratories using an Agilent 6460 LC-MS/MS system (Agilent Technologies, Inc., Santa Clara, CA, USA). The analytical method for the detection of DON, DOM-1, ZEN, α-ZEL, β-ZEL, α-ZAL, β-ZAL, and ZAN in the muscle, kidney, and liver samples was developed and validated in-house. The LOQ (µg/kg) for DON and DOM-1 was 1.6 ppb; for ZEN, it was 0.4 ppb; and for α-ZEL, β-ZEL, α-ZAL, β-ZAL, and ZAN, it was 4 ppb. The method was performed using the following internal standards: [^13^C_15_] DON CRM Biopure^TM^—[25 µg/mL] for DON and DOM-1 and [^13^C_18_] ZEN; CRM Biopure^TM^—[25 µg/mL] for ZEN, α-ZAL, β-ZAL, α-ZEL, β-ZEL, and ZAN toxins. More than 75% recovery was recorded for all toxins. The method was linear from 1.6 to 32 ppb for DON and DOM-1, 0.4 to 8.0 ppb for ZEN, and 4 to 80 ppb for α-ZAL, β-ZAL, α-ZEL, β-ZEL, and ZAN.

Briefly, the tissue samples were lyophilized, finely ground, and thoroughly mixed using a blender, and subsequently, 2 g test portion was removed for analysis. A 10 mL extraction mixture (80% acetonitrile: 19% Water: 1% formic acid) was added to the test portion and then shaken in an orbital shaker at 200× *g* for 1 h at room temperature. After extraction, this portion was centrifuged at 4200× *g* for 5 min, and 7 mL of the supernatant was removed. The sample extract was cleaned by adding 2.8 g of MgSO_4_ and 0.7 g of NaCl to the supernatant, and it was subsequently vortexed for 60 s. The centrifuging of these tubes at 4200× *g* for 5 min followed afterward. A 1 mL solution was removed from the supernatant and diluted with 200 µL of water. Further clean-up was performed on Captiva EMR—Lipid (3 mL, 300 mg) cartridge, (no cartridge conditioning is required), passed through by gravity, and collected into a 15 mL centrifuge tube. When all the extract passed through the cartridge, 500 µL of the sample was evaporated in the evaporator (CHRIST RVC 2-18 CDplus) at 1500× *g* under a 40 °C temperature. Exactly 500 µL of the solvent for reconstitution (50% acetonitrile: 50% water; containing 0.1% formic acid) was added to the evaporated sample, and vortexed. The reconstituted samples were filtered across a nylon membrane syringe (pore size of 0.22 µm) and vortexed. The samples were run on LC-MS/MS (6460c Triple Quad LC/MS, Agilent Technologies) using analytical column Waters CORTECS^®^ UPLC C18 2.1 × 100 mm 1.6 µm and CORTECS^®^ UHPLC C18 2.1 × 5 mm 1.6 µm VanGuard™, UHPLC guard column using the conditions demonstrated in Table A1 (Appendix A). Furthermore, the MS/MS parameters are presented in Table A2, and the retention times of the analytes are shown in Table A3. The validation parameters are presented in Table A4.

The determination of the mycotoxin mass fraction was calculated as follows:

$$C\;\mathrm{Mycotoxin}\;[µg/\mathrm{kg}]=\frac{C*Vr*V}{m}$$

where

C—determined concentration of mycotoxin [ng/mL]
Vr—reconstitution volume (0.5) [mL]
V—acetonitrile volume (8 mL) in the extraction portion
m—amount of sample [g]

### 4.3. Health and Performance Indicators

The primary zootechnical parameters which were calculated during the study included the mean pen BW measured weekly, as well as the mean pen feed intake, mean BWG, and FCR as a mean feed-to-gain ratio, which were calculated in weekly intervals, and for the time periods from day 0 to day 14, from day 15 to day 42, and the total trial period from day 0 to day 42. BWG was calculated using the mean BW per pen at the end of each period minus the mean BW per pen at the start of each period. The ADG was calculated by dividing BWG per period by the number of piglets per pen. The feed consumption per piglet was estimated as the total feed supplied per pen, the period corrected for dispersed or leftover feed, and the number of piglets per pen. FCR was calculated from the relationship between weekly corrected feed intake and growth per piglet for that period.

The daily clinical evaluation of the animals included a fecal consistency assessment, and the respective daily fecal scores were calculated based on the following scoring system: score 0: well-formed feces, firm to cut; 1: pasty feces without falling out of shape; 2: pasty feces falling out of shape upon contact with surfaces; 3: liquid diarrhea.

### 4.4. Hematological and Biochemical Analysis of Blood Samples

Blood samples were taken on day 42 of the trial from one piglet per pen (seven piglets/treatment), which were selected based on the gender and body weights that were closest to the average of the corresponding treatment group. The samples were collected from the anterior vena cava vein into heparinized and EDTA or citrate-containing tubes. The samples were centrifugated at 2000× *g* for 15 min at room temperature. After centrifugation, the plasma was placed into a polypropylene tube and stored at −20 °C until further analysis. The samples were analyzed for hematological and biochemical variables (erythrocytic and leukocytic values: lymphocytes, monocytes, eosinophils, basophils, neutrophils, hemoglobin, hematocrit, mean corpuscular volume (MCV), mean corpuscular hemoglobin (MCH), and mean corpuscular hemoglobin concentration (MCHC). Moreover, platelet counts, prothrombin time, electrolytes (sodium, potassium, chlorine, calcium, magnesium, and inorganic phosphate), biochemical values (total cholesterol, triglycerides, bilirubin, urea, glucose, albumin, globulin, total protein, and creatinine) and enzyme activities [alanine aminotransferase (ALT), alkaline phosphatase (ALP), aspartate-amino-transferase (AST), and glutamate-dehydrogenase (GLDH)] were measured. The selected indicators of the antioxidative response [superoxide dismutase (SOD); malondialdehyde (MDA)], immune response [haptoglobin (HP); C reactive protein (CRP), fibrinogen], and stress response (corticosterone) were determined. In addition, the fecal samples of the piglets used for blood sampling were collected to measure stress (cortisol) and inflammatory biomarkers (calprotectin).

Additional blood testing was carried out by an accredited laboratory (SYNLAB.vet GmbH, Berlin), where blood cells were measured via flow cytometry, and sodium, potassium, and chlorides were measured via ionic liquid-polyacrylamide gel electrophoresis. The immune, oxidative, and gastrointestinal integrity of relevant parameters were tested according to the institute’s (Institute of Animal Nutrition, Freie Universität Berlin) own protocols: namely for calprotectin: Mybiosource: MBS033848 “URL: https://www.mybiosource.com/cp-porcine-elisa-kits/calprotectin/3384 (accessed on 10 March 2025)”; for cortisol: Tecan/IBL: RE52061 “URL: https://ibl-international.com/media/mageworx/downloads/attachment/file/r/e/re52061_ifu_us_en_cortisol_elisa_2023-06_sym9.pdf (accessed on 10 March 2025)”; for GPx: Cayman: 703102 “URL: https://www.caymanchem.com/product/703102/glutathione-peroxidase-assay-kit (accessed on 10 March 2025)” and for SOD: Invitrogen: EIASODC “URL: https://www.thermofisher.com/document-connect/document-connect.html?url=https://assets.thermofisher.com/TFS-Assets%2FLSG%2Fmanuals%2FMAN0019052_EIASODC_SOD_color_activity_PI.pdf (accessed on 10 March 2025)”. 

### 4.5. Statistical Analysis

All data were analyzed by analysis of variance according to a completely randomized design using the software package SPSS (IBM SPSS Version 25). The pen was considered the replicate for performance parameters. Individual piglets were considered the replicate for blood and tissue analysis. All parameters were reported as group least squares mean. Standard error of the mean, difference in the mean, and 95% confidence intervals were also considered. Multiple comparisons between treatment groups were made by Tukey’s and significant differences were declared at *p* ≤ 0.05, while near significant trends were considered for 0.05 < *p* ≤ 0.10. In the case of outliers, the data were not removed prior to statistical analysis.

The experiment was designed to be optimal or near-optimal in the trade-off between power and resources while adhering to the principles of the 3Rs (i.e., to reduce the number of animals used in scientific experiments). Therefore, the number of piglets used in this study was within the range of values that give an appropriate probability (power) of the objectives of the experiment being met; thus, a 6.7% BWG difference between treatments regarding contaminated diets with or without the addition of MMDA is statistically significant (*p* < 0.05). The calculation was based on the *n* = f(α/2, β) × 2 × σ^2^ / (μ1 − μ2)^2^ equation, where μ1 and μ2 are the mean outcomes in the control and experimental group, respectively, σ is the standard deviation, and f(α, β) = [Φ^−1^(α) + Φ^−1^(β)]^2^, where Φ^−1^ is the cumulative distribution function of a standardized normal deviate.

## Figures and Tables

**Table 1:** Performance parameters of trial groups in the starter, grower, and overall feeding period of the trial (day 25 to day 66 of age) presented as the means of seven pens/treatment (six piglets/pen) ± standard deviation (SD).

Trial Groups ^∍^					
Parameters	T1	T2	T3	T4	*p*-Values
Starter feeding period: day 0 to day 14 of the trial period (day 25 to day 38 of age)					
BW * on d 0 (kg)	6.93 ± 0.89	6.94 ± 0.92	6.94 ± 0.89	6.94 ± 0.89	1.000
BW on d 14 (kg)	12.17 ± 1.11	10.92 ± 0.86	11.64 ± 1.07	11.84 ± 0.99	0.153
BWG * (kg)	5.24 ± 0.43 ^a^	3.98 ± 0.56 ^b^	4.71 ± 0.04 ^ab^	4.91 ± 0.60 ^b^	0.022
ADG *	374 ± 31 ^a^	284 ± 40 ^b^	336 ± 43 ^ab^	351 ± 43 ^b^	0.022
Feed intake (kg)	7.30 ± 0.93	6.14 ± 0.93	6.88 ± 0.97	6.98 ± 0.99	0.163
ADFI *	522 ± 66	438 ± 66	491 ± 69	498 ± 70	0.163
FCR *	1.390 ± 0.089	1.542 ± 0.061	1.468 ± 0.165	1.422 ± 0.110	0.094
Grower feeding period: day 15 to day 42 of the trial period (day 39 to day 66 of age)					
BW on d 42 (kg)	33.56 ± 1.40 ^a^	29.46 ± 0.82 ^b^	30.97 ± 1.43 ^b^	33.49 ± 1.48 ^a^	<0.001
BWG (kg)	21.38 ± 0.97 ^a^	18.54 ± 0.34 ^b^	19.33 ± 0.63 ^b^	21.64 ± 0.79 ^a^	<0.001
ADG	764 ± 35 ^a^	662 ± 12 ^b^	690 ± 23 ^b^	773 ± 28 ^a^	<0.001
Feed intake (kg)	31.41 ± 1.02 ^c^	29.50 ± 0.98 ^ab^	28.69 ± 0.99 ^a^	30.81 ± 1.45 ^bc^	<0.001
ADFI	1.12 ± 0.04 ^c^	1.05 ± 0.04 ^ab^	1.02 ± 0.04 ^a^	1.10 ± 0.05 ^bc^	<0.001
FCR	1.470 ± 0.040 ^a^	1.592 ± 0.061 ^b^	1.485 ± 0.051 ^a^	1.424 ± 0.037 ^a^	<0.001
Overall feeding period: day 0 to day 42 of the trial period (day 25 to day 66 of age)					
BW on d 0 (kg)	6.93 ± 0.89	6.94 ± 0.92	6.94 ± 0.89	6.94 ± 0.89	1.000
BW on d 42 (kg)	33.56 ± 1.40 ^a^	29.46 ± 0.82 ^b^	30.97 ± 1.43 ^b^	33.49 ± 1.48 ^a^	<0.001
BWG (kg)	26.62 ± 0.80 ^c^	22.52 ± 0.43 ^a^	24.03 ± 0.83 ^b^	26.55 ± 0.64 ^c^	<0.001
ADG	634 ± 19 ^c^	536 ± 10 ^a^	572 ± 20 ^b^	632 ± 15 ^c^	<0.001
Feed intake (kg)	38.71 ± 1.11 ^b^	35.64 ± 1.27 ^a^	35.57 ± 1.74 ^a^	37.79 ± 1.42 ^b^	<0.001
ADFI	922 ± 26 ^b^	849 ± 30 ^a^	847 ± 42 ^a^	900 ± 34 ^b^	<0.001
FCR	1.455 ± 0.039 ^a^	1.583 ± 0.052 ^b^	1.480 ± 0.039 ^a^	1.423 ± 0.032 ^a^	<0.001

* BW: Body weight; BWG: body weight gain; ADG: average daily gain; ADFI: average daily feed intake; FCR: feed-to-gain ratio. ^∍^ The T1 group received feed without mycotoxins or MMDA; group T2 pigs received contaminated feed with 1.5 mg of DON/kg feed and 0.9 mg of ZEN/kg feed for the first two weeks and 1.2 mg of DON/kg feed and 0.9 mg of ZEN/kg feed for the rest of the trial period, without the addition of MMDA; groups T3 and T4 received the same DON and ZEN contaminated feed as the T2 group with the addition of 1.5 g of MMDA/kg feed (T3), or 3 g of MMDA/kg feed (T4). ^a,b,c^ Means with different superscripts in the same row differ significantly (*p* ≤ 0.05).

**Table 2:** Blood profiles of piglets (*n* = seven animals per group) at the end of the study (day 66 of age) as a means ± standard deviation (SD).

	Trial Groups ^∍^					
Parameters	T1	T2	T3	T4	*p* Values	Normal Limits
Blood constituents						
Erythrocytes (G/L)	6.06 ± 0.15	5.48 ± 0.34	5.88 ± 0.88	5.89 ± 1.20	0.544	5.8–8.1
Leukocytes (G/L)	20.54 ± 3.72	16.41 ± 2.44	19.09 ± 3.50	18.49 ± 2.48	0.121	11–22
Platelets (G/L)	127.3 ± 21.7	109.9 ± 42.9	130.3 ± 52.7	132.7 ± 51.2	0.761	220–620
Lymphocytes (L; %)	46.3 ± 5.0 ^a^	51.0 ± 4.4 ^ab^	53.9 ± 4.8 ^b^	47.3 ± 5.9 ^ab^	0.037	49–85
H/L ratio	0.94 ± 0.09	0.97 ± 0.10	0.80 ± 0.16	0.84 ± 0.14	0.055	
Monocytes (%)	1.4 ± 0.8 ^a^	3.3 ± 1.1 ^b^	1.4 ± 0.8 ^a^	2.0 ± 1.6 ^ab^	0.016	0–5
Eosinophils (%)	2.1 ± 0.9	2.6 ± 0.5	2.0 ± 1.3	2.6 ± 1.0	0.588	0–6
Basophils (%)	0.4 ± 0.5	0.4 ± 0.5	0.3 ± 0.5	0.4 ± 0.5	0.941	0–2
Hemoglobin (g/L)	112.7 ± 5.9	109.9 ± 6.2	112.4 ± 19.4	113.3 ± 21.1	0.974	100–160
Haematocrit (l/L)	0.40 ± 0.02	0.37 ± 0.03	0.37 ± 0.06	0.38 ± 0.07	0.700	0.33–0.45
MCV ^1)^ (Fl)	65.2 ± 2.4	66.8 ± 4.7	63.5 ± 3.2	64.6 ± 6.0	0.541	50–70
MCH ^2)^ (Pg)	18.6 ± 0.7	20.1 ± 0.7	19.1 ± 1.0	19.4 ± 1.6	0.091	17–22
MCHC ^3)^ (g/dL)	28.6 ± 0.6 ^a^	30.1 ± 1.3 ^b^	30.0 ± 1.0 ^b^	30.1 ± 1.3 ^b^	0.030	30–35
Electrolytes and enzyme activities						
Sodium (mmol/L)	142.1 ± 1.1	142.3 ± 1.5	143.9 ± 1.3	142.4 ± 2.7 ^b^	0.263	135–160
Potassium (mmol/L)	4.2 ± 0.4	4.1 ± 0.2	4.3 ± 0.3	4.2 ± 0.4	0.837	4–5
Chloride (mmol/L)	103.3 ± 2.0	101.9 ± 1.8	102.6 ± 1.9	102.1 ± 2.0	0.535	102–106
Calcium (mmol/L)	2.7 ± 0.1	2.6 ± 0.2	2.5 ± 0.1	2.8 ± 0.1	0.070	1.8–2.9
Inorganic phosphate (mmol/L)	3.0 ± 0.4	3.1 ± 0.4	3.1 ± 0.3	2.9 ± 0.4	0.789	1.9–3.2
AST ^4)^ (U/L)	51± 5 ^a^	86 ± 26 ^c^	77 ± 14 ^bc^	60 ± 8 ^ab^	0.001	29–87
ALT ^5)^ (U/L)	50 ± 11	58 ± 10	46 ± 14	54 ± 18	0.444	<113
GLDH ^6)^ (U/L)	<2.0	<2.0	<2.0	<2.0		<6.4
AP ^7)^ (U/L)	240 ± 48	293 ± 63	254 ± 57	253 ± 30	0.263	<280
SOD ^8)^ (U/mL)	0.69 ± 0.12	1.06 ± 0.06	1.16 ± 0.32	0.72 ± 0.13	0.079	(0.5–3.0)
Blood metabolites						
Total cholesterol (mmol/L)	2.1 ± 0.5	1.9 ± 0.5	1.8 ± 0.3	2.0 ± 0.7	0.778	1.5–3.3
Triglycerides (mmol/L)	0.3 ± 0.1	0.2 ± 0.1	0.3 ± 0.1	0.3 ± 0.1	0.128	<0.83
Urea (mmol/L)	4.1 ± 0.3	4.8 ± 0.7	4.1 ± 0.9	4.0 ± 0.3	0.065	2.5–6.7
Total bilirubin (μmol/L)	1.6 ± 0.6	2.3 ± 0.3	1.8 ± 0.5	1.6 ± 0.9	0.125	<4.5
Glucose (mmol/L)	6.9 ± 1.3	7.7 ± 1.0	7.2 ± 1.3	7.0 ± 1.3	0.619	4.05–6.6
Albumins (mmol/L)	22.0 ± 1.1	20.1 ± 1.5	21.0 ± 1.5	21.2 ± 1.0	0.070	27–42
Globulins (mmol/L)	28.8 ± 3.6	27.6 ± 4.1	26.4 ± 4.2	32.2 ± 4.4	0.072	18–43
Total protein (mmol/L)	50.8 ± 3.6 ^ab^	47.7 ± 3.2 ^ab^	47.4 ± 4.6 ^a^	53.4 ± 4.4 ^b^	0.029	45–85
Haptoglobin (mg/mL)	0.41 ± 0.09 ^ab^	0.49 ± 0.09 ^a^	0.40 ± 0.09 ^ab^	0.35 ± 0.05 ^b^	0.029	<1
CRP ^9)^ (μg/mL)	15.5 ± 3.9	17.2 ± 1.9	15.7 ± 3.8	15.4 ± 2.6	0.695	(10–25) *
Corticosterone (pg/mL)	84.7 ± 23.6	130.0 ± 47.6	101.3 ± 58.0	97.0 ± 32.9	0.264	(66–200) *
MDA ^10)^ (nmol/L)	3.4 ± 0.3 ^b^	4.1 ± 0.5 ^c^	3.5 ± 0.4 ^b^	2.8 ± 0.3 ^a^	<0.001	(2–5) *
Fibrinogen (g/L)	2.2 ± 0.4	2.7 ± 0.8	1.9 ± 0.6	2.1 ± 0.4	0.067	(1.5–3)^2^
Prothrombin time (sec)	13.5 ± 0.8	14.1 ± 1.0	13.5 ± 0.6	13.3 ± 0.7	0.298	11–15

^1)^ Mean corpuscular volume; ^2)^ mean corpuscular hemoglobin; ^3)^ mean corpuscular hemoglobin concentration; ^4)^ aspartate transaminase, ^5)^ alanine aminotransferase; ^6)^ glutamate dehydrogenase; ^7)^ alkaline phosphatase; ^8)^ superoxide dismutase; ^9)^ C-reactive protein; ^10)^ malondialdehyde. * Institute (Institute of Animal Nutrition, Freie Universität Berlin) own reference values. ^∍^ The T1 group received feed without mycotoxins or MMDA; the pigs in group T2 received contaminated feed with 1.5 mg DON/kg feed and 0.9 mg of ZEN/kg feed for the first two weeks, and 1.2 mg of DON/kg feed and 0.9 mg of ZEN/kg feed for the rest of the trial period, without the addition of MMDA; groups T3 and T4 received the same DON- and ZEN-contaminated feed as the T2 group, with the addition of 1.5 g of MMDA/kg feed (T3), or 3 g of MMDA/kg feed (T4). ^a,b,c^ Means with different superscripts in the same row differ significantly (*p* ≤ 0.05).

**Table 3:** Levels of calprotectin and cortisol in feces (*n* = seven animals per group) as a means ± standard deviation (SD) at the end of the study period (day 66 of age).

Treatment Groups ^∍^					
Parameters (ng/g)	T1	T2	T3	T4	*p* Value
Stress biomarker on day 66 of age (day 42 on trial)					
Cortisol	17.17 ± 1.92 ^a^	25.19 ± 2.96 ^c^	22.07 ± 2.86 ^bc^	19.68 ± 2.87 ^ab^	<0.001
Calprotectin	746.71 ± 28.16 ^a^	794.70 ± 24.82 ^b^	782.49 ± 33.40 ^ab^	765.72 ± 34.87 ^ab^	0.039

**^∍^** T1 group received feed without mycotoxins or MMDA; the pigs in group T2 received contaminated feed with 1.5 mg of DON/kg feed and 0.9 mg of ZEN/kg feed for the first two weeks, and 1.2 mg of DON/kg feed and 0.9 mg of ZEN/kg feed for the rest of the trial period, without the addition of MMDA; groups T3 and T4 received the same DON- and ZEN-contaminated feed as the T2 group, with the addition of 1.5 g of MMDA/kg feed (T3), or 3 g of MMDA/kg feed (T4). ^a,b,c^ Means with different superscripts in the same row differ significantly (*p* ≤ 0.05).

**Table 4:** Mycotoxin residues in tissues (*n* = seven animals per group) at the end of the study period (day 66 of age) as means ± standard deviation (SD).

Treatment Groups ^∍^					
Mycotoxin (μg/kg)	T1	T2	T3	T4	*p* Value
Liver samples on day 66 of age (day 42 on trial)					
DON	<1.60 ^a^	7.23 ± 1.79 ^b^	6.17 ± 2.51 ^b^	6.02 ± 2.20 ^b^	<0.001
ZEN	<0.40 ^a^	1.10 ± 0.26 ^b^	1.06 ± 0.36 ^b^	1.00 ± 0.31 ^b^	<0.001
Kidney samples on day 66 of age (day 42 on trial)					
DON	<1.60 ^a^	60.23 ± 17.93 ^c^	34.33 ± 17.78 ^b^	32.34 ± 26.25 ^b^	<0.001
ZEN	<0.40 ^a^	1.43 ± 0.27 ^b^	1.23 ± 0.47 ^b^	1.21 ± 0.44 ^b^	<0.001

**^∍^** T1 group received feed without mycotoxins or MMDA; the pigs in group T2 received contaminated feed with 1.5 mg of DON/kg feed and 0.9 mg of ZEN/kg feed for the first two weeks and 1.2 mg of DON/kg feed and 0.9 mg of ZEN/kg feed for the rest of the trial period, without the addition of MMDA; groups T3 and T4 received the same DON- and ZEN-contaminated feed as the T2 group, with the addition of 1.5 g of MMDA/kg feed (T3), or 3 g of MMDA/kg feed (T4). ^a,b,c^ Means with different superscripts in the same row differ significantly (*p* ≤ 0.05).

**Table 5:** Composition and analysis of the starter diets from day 25 to day 38 of age (as-fed).

Treatment Groups	T1	T2	T3	T4
Ingredients (%)				
Wheat	48.61	48.61	48.61	48.61
Soybean meal (CP: 49%)	16.59	16.59	16.59	16.59
Barley	18.00	18.00	18.00	18.00
Skim milk powder	10.00	10.00	10.00	10.00
Soybean oil	1.40	1.40	1.40	1.40
Limestone	1.55	1.55	1.55	1.55
Minerals and vitamins ^1)^	1.20	1.20	1.20	1.20
Monocalcium phosphate	0.90	0.90	0.90	0.90
L-lysine-HCL	0.55	0.55	0.55	0.55
DL-methionine	0.17	0.17	0.17	0.17
L-threonine	0.19	0.19	0.19	0.19
L-tryptophan	0.04	0.04	0.04	0.04
Tixosil ^2)^	0.30	0.30	0.15	
MMDA			0.15	0.30
Maize control	0.34			
Maize control + DON and ZEN		0.34	0.34	0.34
Calculated analysis (%, except ME)				
ME ^3)^ (MJ/kg)	13.40	13.40	13.40	13.40
Crude protein	20.00	20.00	20.00	20.00
Lysine	1.45	1.45	1.45	1.45
Methionine	0.51	0.51	0.51	0.51
Methionine and cysteine	0.84	0.84	0.84	0.84
Threonine	0.92	0.92	0.92	0.92
Tryptophan	0.28	0.28	0.28	0.28
SID Lysine	1.25	1.25	1.25	1.25
SID methionine	0.46	0.46	0.46	0.46
SID threonine	0.70	0.70	0.70	0.70
SID tryptophan	0.23	0.23	0.23	0.23
Crude fat	2.96	2.96	2.96	2.96
Crude fiber	2.88	2.88	2.88	2.88
Crude ash	5.78	5.78	5.78	5.78
Calcium	0.95	0.95	0.95	0.95
Available phosphorus	0.44	0.44	0.44	0.44
Sodium	0.25	0.25	0.25	0.25
Analyzed nutrient levels (g/kg)				
Dry matter	904.0	903.1	903.7	904.1
Crude protein	203.7	204.3	205.1	204.2
Crude fiber	29.1	29.6	30.0	30.2
Crude ash	59.2	58.8	58.7	58.9
Crude fat	31.1	30.9	31.2	31.0
Starch	326.4	327.0	327.1	326.8
Total sugars	97.3	96.7	96.9	97.2
Calcium	9.4	9.5	9.4	9.5
Phosphorus	7.1	7.0	7.2	7.0
Sodium	1.9	1.9	1.8	1.9
Mycotoxin contamination levels (μg/kg feed)				
Alflatoxin	2.0	1.9	1.9	2.0
DON	127	1475	1482	1497
T2/HT2	10	11	12	11
Ochratoxin	<1	<1	<1	<1
ZEN	46	926	930	931

^1)^ Contents per kg of premix: 400,000 I.U. vit. A (acetate); 120,000 I.U. vit. D_3_; 8000 mg of vit. E (α-tocopherole acetate); 200 mg of vit. K_3_ (MSB); 250 mg of vit. B_1_ (mononitrate); 420 mg of vit. B_2_ (cryst. riboflavin); 2500 mg of niacin (niacinamide); 400 mg of Vit. B_6_ (HCl); 2000 μg of vit. B_12_; 25,000 μg of biotin (commercial feed grade); 1000 mg of pantothenic acid (Ca d-pantothenate); 100 mg of folic acid (cryst. commercial feed grade); 80,000 mg of choline (chloride); 5000 mg Zn (sulfate); 5000 mg of Fe (carbonate); 6000 mg of Mn (sulfate); 1000 mg of Cu (sulfate–entahydrate); 20 mg of Se (Na-selenite); 45 mg of J (Ca-iodate); 130 g of Na (NaCl); 55 g of Mg (sulfate); ^2)^ silicon dioxide > 97%. ^3)^ All measurements were calculated by using the estimation given by DLG [84].

**Table 6:** Composition and analysis of the grower diets from 39 to 66 days of age (as-fed).

Treatment Groups	T1	T2	T3	T4
Ingredients (%)				
Wheat	49.94	49.94	49.94	49.94
Barley	22.00	22.00	22.00	22.00
Soybean meal (CP: 49%)	20.62	20.62	20.62	20.62
Soybean oil	2.10	2.10	2.10	2.10
Limestone	1.56	1.56	1.56	1.56
Minerals and vitamins ^1)^	1.20	1.20	1.20	1.20
Monocalcium phosphate	1.16	1.16	1.16	1.16
L-lysine-HCl	0.50	0.50	0.50	0.50
L-threonine	0.14	0.14	0.14	0.14
DL-methionine	0.12	0.12	0.12	0.12
L-tryptophan	0.02	0.02	0.02	0.02
Tixosil ^2)^	0.30	0.30	0.15	
MMDA			0.15	0.30
Maize control	0.34			
Maize control + DON and ZEN		0.34	0.34	0.34
Calculated analysis (%, except ME)				
ME ^3)^ (MJ/kg)	13.40	13.40	13.40	13.40
Crude protein	19.00	19.00	19.00	19.00
Lysine	1.30	1.30	1.30	1.30
Methionine	0.41	0.41	0.41	0.41
Methionine and cysteine	0.75	0.75	0.75	0.75
Threonine	0.82	0.82	0.82	0.82
Tryptophan	0.25	0.25	0.25	0.25
SID lysine	1.12	1.12	1.12	1.12
SID methionine	0.36	0.36	0.36	0.36
SID threonine	0.63	0.63	0.63	0.63
SID tryptophan	0.21	0.21	0.21	0.21
Crude fat	3.76	3.76	3.76	3.76
Crude fiber	3.27	3.27	3.27	3.27
Crude ash	5.63	5.63	5.63	5.63
Calcium	0.88	0.88	0.88	0.88
Available phosphorus	0.42	0.42	0.42	0.42
Sodium	0.21	0.21	0.21	0.21
Analyzed nutrient levels (g/kg)				
Dry matter	901.5	901.0	901.6	901.2
Crude protein	198.4	198.0	198.4	197.8
Crude fiber	34.2	34.0	33.7	34.5
Crude ash	57.6	58.0	57.4	58.2
Crude fat	38.9	39.2	39.4	39.0
Starch	347.2	346.8	347.3	347.9
Total sugars	88.5	89.3	88.6	90.1
Calcium	8.9	8.9	9.0	8.9
Phosphorus	6.6	6.5	6.6	6.7
Sodium	1.9	2.0	1.9	1.8
Mycotoxin contamination levels (µg/kg)				
Alflatoxin	2.2	2.0	2.1	2.2
DON	156	1171	1162	1173
T2/HT2	12	13	11	12
Ochratoxin	<1	<1	<1	<1
ZEN	53	868	873	870

^1)^ Contents per kg of premix: 400,000 I.U. vit. A (acetate); 120,000 I.U. vit. D_3_; 8000 mg of vit. E (α-tocopherole acetate); 200 mg of vit. K_3_ (MSB); 250 mg of vit. B_1_ (mononitrate); 420 mg of vit. B_2_ (cryst. riboflavin); 2500 mg of niacin (niacinamide); 400 mg of Vit. B_6_ (HCl); 2000 μg of vit. B_12_; 25,000 μg of biotin (commercial feed grade); 1000 mg of pantothenic acid (Ca d-pantothenate); 100 mg of folic acid (cryst. commercial feed grade); 80,000 mg of choline (chloride); 5000 mg of Zn (sulfate); 5000 mg of Fe (carbonate); 6000 mg of Mn (sulfate); 1000 mg of Cu (sulfate–entahydrate); 20 mg of Se (Na-selenite); 45 mg of J (Ca-iodate); 130 g of Na (NaCl); 55 g mg of (sulfate); ^2)^ silicon dioxide > 97%. ^3)^ All measurements were calculated by using the estimation given by DLG [84].

## Data Availability

The original contributions presented in this study are included in the article/supplementary material. Further inquiries can be directed to the corresponding authors.

## Supplementary Materials

The following supporting information can be downloaded at: https://www.mdpi.com/article/10.3390/toxins17030146/s1, Figure S1: Chromatograms of ZEN residues in kidney samples of each trial group at the end of the trial period; Figure S2: Chromatograms of ZEN residues in liver samples of each trial group at the end of the trial period; Figure S3: Chromatograms of DON residues in kidney samples of each trial group at the end of the trial period; Figure S4: Chromatograms of DON residues in liver samples of each trial group at the end of the trial period.

## Appendix A

The details of methodology and validation parameters used for the mycotoxin residues analysis in tissues are presented in the following Table A1, Table A2, Table A3 and Table A4.

**Table A1:** UPLC gradient conditions.

Time [min]	Mobil Phase A [%]	Mobile Phase B [%]	Flow [mL/min]
0.00	95.00	5.00	0.300
4.00	80.00	20.00	0.300
9.00	80.00	20.00	0.300
15.00	20.00	80.00	0.300
17.00	20.00	80.00	0.300
18.00	0.00	100.00	0.300
21.00	0.00	100.00	0.300
22.00	95.00	5.00	0.300

**Table A2:** MS/MS parameters.

Gas Temperature [°C]	200
Gas Flow [L/min]	8
Nebulizer [psi]	40
Sheath Gas Temp [°C]	350
Sheath Gas Flow [L/min]	11
Capillary [V]	3500
Nozzle Voltage [V]	500

**Table A3:** MRM transitions, MS/MS parameters, and retention times of the analyses.

Compound Name	Precursor Ion	Product Ion	Fragmentor (V)	Collision Energy (V)	Polarity	Retention Time, min
[^13^C_15_] DEOXYNIVALENOL	312.2	263.1	84	8	Positive	4.36
		245.1		12		
DEOXYNIVALENOL	297.1	248.8	100	9	Positive	4.36
		230.8		9		
DOM-1	281.1	137	128	12	Positive	6.20
		109		16		
[^13^C_18_] ZEARALENONE	335.2	185	176	20	Negative	16.20
		169		32		
α—ZEARALENOL	319.2	160	186	28	Negative	15.91
		130		36		
β—ZEARALENOL	319.2	160	186	28	Negative	15.41
		130		36		
α—ZEARALANOL	321.2	303.1	150	20	Negative	15.78
		277.1		20		
β—ZEARALANOL	321.2	303.1	128	16	Negative	15.25
		277.1		20		
ZEARALANONE	319.2	275.1	146	16	Negative	16.15
		205		20		
ZEARALENONE	317.1	175	190	20	Negative	16.20
		131.1		28		

**Table A4:** Validation parameters of DON, ZEN, and metabolites.

	Analyte							
Validation Parameters	DON	DOM-1	ZEN	ZAN	α-ZEL	β-ZEL	α-ZAL	β-ZAL
Linear range; ng/mL	0.8–16	0.8–16	0.2–4.0	2.0–40.0	2.0–40.0	2.0–40.0	2.0–40.0	2.0–40.0
Linearity, R^2^	0.999	0.997	0.998	0.996	0.998	0.998	0.999	0.999
Recovery, %	79.9	75.6	95.1	92.6	90.2	89.8	89.9	90.2
LOQ (μg/kg)	1.60	1.60	0.40	4.0	4.0	4.0	4.0	4.0
LOD (μg/kg)	0.50	0.50	0.15	1.5	1.5	1.5	1.5	1.5
