# Supplementary material for: Effects of a Multicomponent Mycotoxin Detoxifying Agent on Health and Performance of Weaned Pigs Under Combined Dietary Exposure to Deoxynivalenol (DON) and Zearalenone (ZEN)
Source: Toxins (Basel). 2025 Mar 19;17(3):146. doi: 10.3390/toxins17030146 (PMC11946165; doi:10.3390/toxins17030146)
Supplement: Supplementary file 1 [file toxins-17-00146-s001.zip › Figure S2 ZEN residues in liver samples.pdf]

Figure S2. Chromatograms of ZEN residues in liver samples of each trial group at the end of the trial period[3]

Group T1[3]

- MRM (317.1 -> 175.0) S24 01 200 emr.d

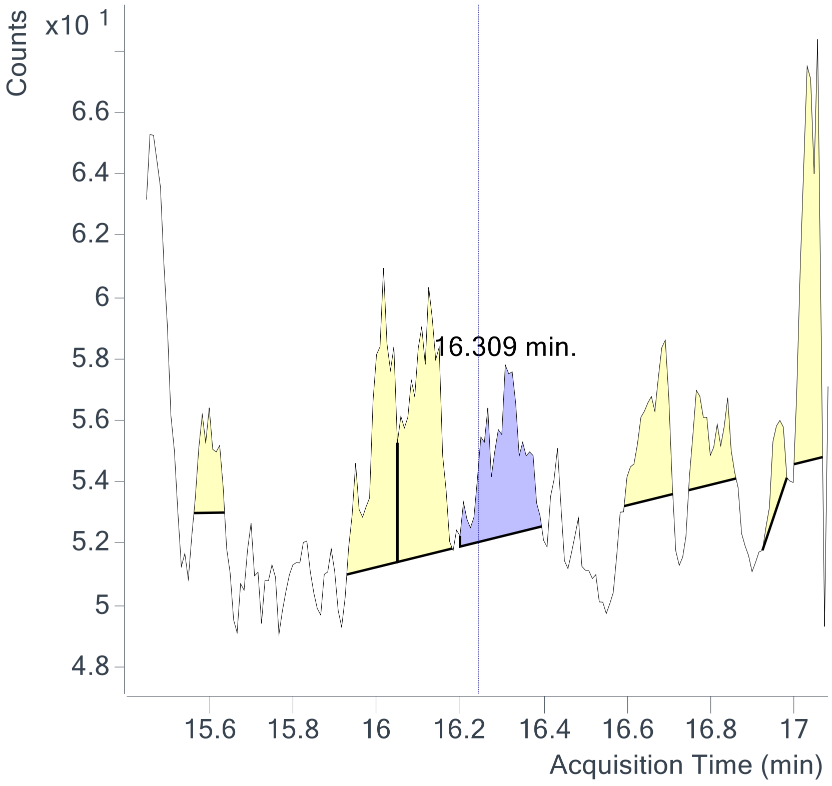

Group T2[3]

- MRM (317.1 -> 175.0) S24 01 209 emr.d

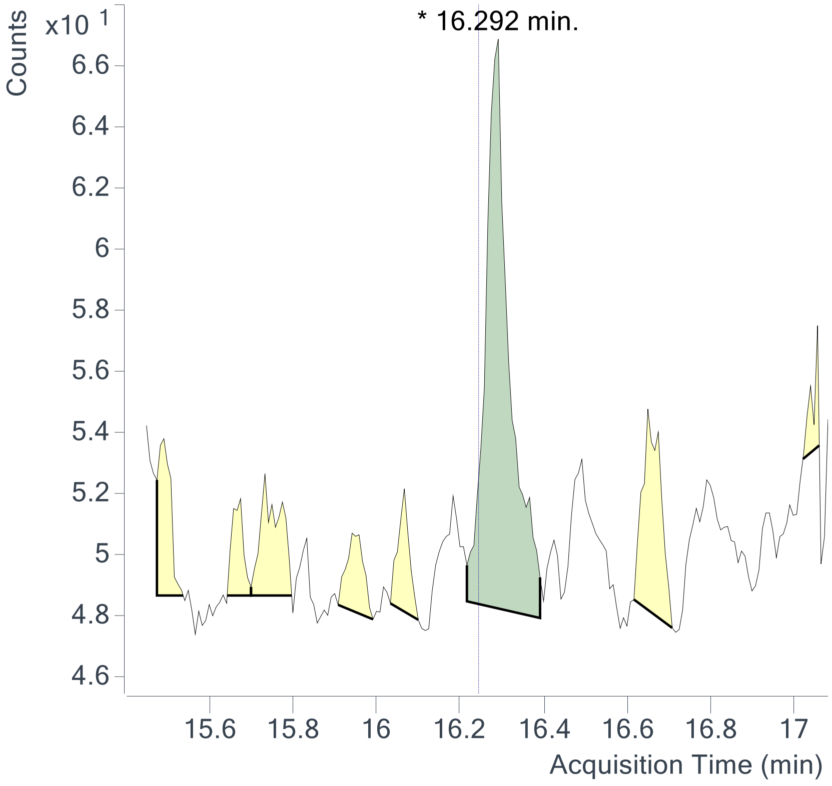

Group T3[ə]

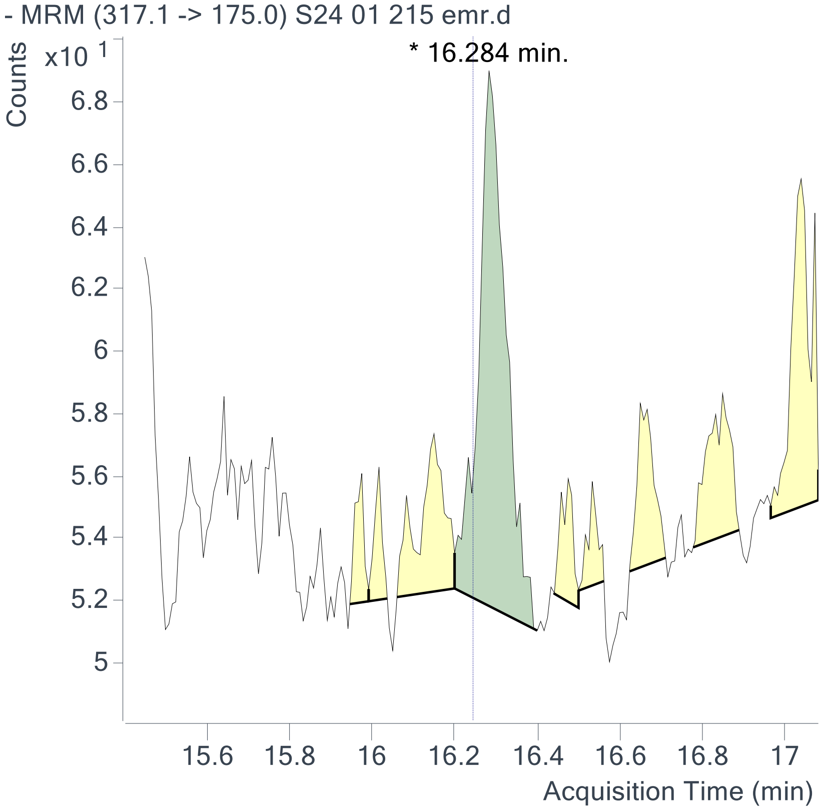

Group T4[ə]

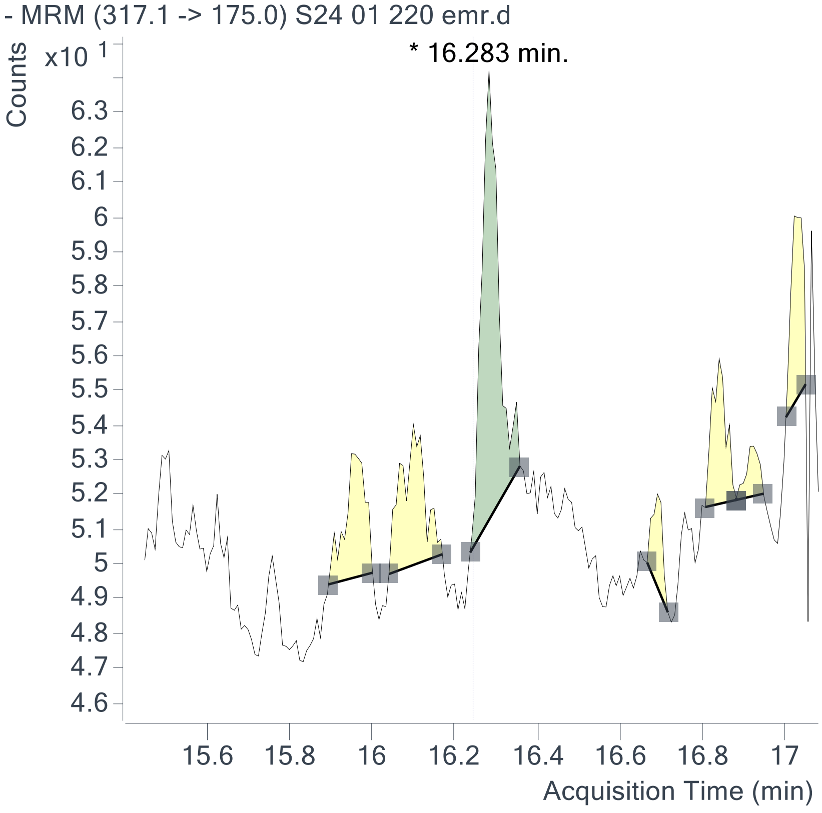

[ə] T1 group received feed without mycotoxins or MMDA; group T2 pigs received contaminated feed with 1.5 mg DON/kg feed and 0.9 mg ZEN/kg feed for the first two weeks and 1.2 mg

DON/kg feed and 0.9 mg ZEN/kg feed for the rest of the trial period, without the addition of MMDA; groups T3 and T4 received the same DON and ZEN contaminated feed as the T2 group, with the addition of 1.5 g MMDA/kg feed (T3), or 3 g MMDA/kg feed (T4).
